# The Clinical and Antibacterial Effects of a Herbal Toothpaste Containing White Mustard *Sinapis alba* Extract: A Randomized Clinical Trial

**DOI:** 10.3390/dj13040165

**Published:** 2025-04-15

**Authors:** Konrad Michałowski, Bartłomiej Górski, Aniela Brodzikowska

**Affiliations:** 1 Department of Conservative Dentistry, Medical University of Warsaw, 02-091 Warsaw, Poland; aniela.brodzikowska@wum.edu.pl; 2 Department of Periodontology and Oral Diseases, Medical University of Warsaw, 02-091 Warsaw, Poland; bgorski@wum.edu.pl

**Keywords:** caries, *Lactobacillus*, mustard, preventive dentistry, plant-based products, *Streptococcus mutans*, toothpaste, oral healthcare

## Abstract

**Objectives:** The aim of this double-blind, clinical trial study was to evaluate the clinical and antibacterial effects of a herbal toothpaste containing white mustard *Sinapis alba* extract in comparison to a placebo toothpaste. **Methods:** One hundred and thirteen participants with gingivitis were randomly assigned to the test group (experimental herbal toothpaste) or the control group (placebo toothpaste). The plaque index (PI), approximal plaque index (API), gingival index (GI), and bleeding on probing (BoP) were evaluated, and salivary samples for microbial evaluation of the loads of *Streptococcus mutans* and *Lactobacillus* spp. were collected at baseline (T_0_) and after 4 weeks (T_1_). Comparisons were performed between and within groups. **Results:** A reduction in all periodontal parameters (PI, API, GI, and BoP) was observed. The experimental toothpaste reduced the PI by 2.43, compared to a 1.95 reduction for the placebo (*p* = 0.041), and BoP by 30.6%, compared to a 26.8% reduction for the placebo (*p* = 0.037). Statistically significant reductions in salivary *S. mutans* and *Lactobacillus* spp. counts were found in the test group. Among patients who used the experimental toothpaste, 19.2% and 9.6% showed counts of *S. mutans* and *Lactobacillus* spp., respectively, below 10^5^ CFU/mL, compared to 44.2% and 40.4% in the placebo group. **Conclusions:** Toothpaste enhanced with white mustard extract was more effective in reducing the PI and BoP indices and decreasing *S. mutans* and *Lactobacillus* spp. counts compared to placebo toothpaste.

## 1. Introduction

Dental caries represents a multifactorial pathological condition characterized by the localized destruction of dental hard tissues through acid-mediated demineralization [1]. It is one of the predominant noncommunicable diseases worldwide, ubiquitously affecting diverse demographic cohorts irrespective of socioeconomic status.

The concentration of cariogenic bacteria in saliva, particularly *Streptococcus mutants* and *Lactobacillus* spp., plays a crucial role in the initiation and progression of dental caries. Higher bacteria loads contribute to an increase in acid production, leading to the demineralization of enamel and dentin. Studies have shown that individuals with salivary *Streptococcus mutans* counts exceeding 10^5^ CFU/mL have a significantly higher risk of caries development. Bacterial concentrations in saliva are dynamic and can change due to factors such as dietary intake, oral hygiene, and the use of antimicrobial agents [2]. Various strategies can effectively reduce cariogenic bacterial loads, including mechanical plaque removal by brushing, the use of antibacterial agents such as chlorhexidine or fluoride, and dietary modifications like limiting fermentable carbohydrates. Additionally, natural antimicrobial compounds, such as plant extracts, have been shown to disrupt bacterial adhesion and inhibit biofilm formation.

Bacteria are aggregated in biofilm. A heterogeneous structure comprising mainly microbial cells (10–25%) and a self-produced extracellular polymeric substance (EPS) matrix (75–90%), which helps in nutrient cycling, provides the availability of deoxyribonucleic acid (DNA) for horizontal gene transfer (HGT) and acts as a protective barrier [3]. The biofilm formation is a multi-step process governed by external conditions such as temperature, gravitational forces, hydrodynamic forces, pH, nature of the inhabiting surfaces, quorum sensing, nutrient availability, cell-to-cell communication, signaling cascades, and various secondary messengers [4]. The formation of cariogenic biofilm starts with the attachment of planktonic microbial cells to tooth surfaces, followed by microbial division to create microcolonies, which undergo maturation and dispersion [5]. Dental biofilms are characterized by a high level of spatial matrix organization and up to 700 distinct microbial species or phylotypes [6]. *Streptococcus* spp. is considered a common initial colonizer of dental biofilm due to its virulence and ability to process sugar carbohydrates [7]. Glucosyltransferase enzymes (Gtfs) derived from *Streptococcus mutans* (*S. mutans*) use various sugar carbohydrates to produce glucan polymer, which renders the biofilm recalcitrant to antimicrobials and difficult to remove [8]. This phenomenon leads to the dominance of acidogenic and acid-tolerating species in the biofilm. Apart from *Streptococcus* spp, other species with acidogenic phenotypes, such as *Lactobacillus* spp., play vital roles in prolonging periods of low pH in the biofilm [9]. The presence of specific functional ecotypes with an inclination to saccharification or proteolysis may initiate the demineralization of the apatite structures of enamel and dentin, potentially progressing to cavitation if a biochemical assault persists [10].

Preventative strategies for dental caries necessitate a multipronged approach, integrating both population-level and individual-level interventions. Public health measures involve water fluoridation and structured, community-based educational prevention programs [11,12]. On the individual scale, rigorous adherence to oral hygiene practices—brushing with fluoride toothpaste, flossing, and dietary regulation—are paramount. Herbal toothpaste additives offer promising benefits in the prevention of dental caries due to their natural antibacterial, anti-inflammatory, and remineralizing properties [13]. Integrating herbal additives in toothpaste formulations can substantially enhance oral hygiene practices, providing a natural, holistic approach to caries prevention and overall dental health [14,15].

White mustard *Sinapis alba* extract is known for its potent antimicrobial properties, which are primarily attributed to glucosinolates and their hydrolysis products, particularly isothiocyanates like allyl isothiocyanate [16]. When plant tissues are damaged, glucosinolates are hydrolyzed by the enzyme myrosinase, releasing isothiocyanates [17]. These compounds exhibit strong antibacterial effects by disrupting bacterial cell membranes, increasing permeability and causing the leakage of cellular contents, which ultimately leads to cell death. Isothiocyanates also react with thiol groups in bacterial enzymes, inhibiting essential processes such as energy production and cell wall synthesis [18,19,20]. Additionally, they induce oxidative stress by generating reactive oxygen species (ROS), damaging cellular components including DNA, proteins, and lipids [21]. Isothiocyanates inhibit biofilm formation, enhancing bacterial susceptibility to antimicrobial agents [22]. These mechanisms collectively contribute to a reduction in microbial populations, making glucosinolates valuable in natural antibacterial strategies.

Thus, the aim of this study was to evaluate the effectiveness of a newly formulated herbal toothpaste containing white mustard extract in reducing salivary *S. mutans* and *Lactobacillus* spp. loads compared to a standard fluoride-free control toothpaste. The working hypothesis was that a toothpaste that incorporates white mustard extract might be more effective in improving periodontal parameters (PI, API, GI, BoP) and would exhibit stronger antibacterial effects in comparison to a control toothpaste.

## 2. Materials and Methods

### 2.1. Study Design

This study was designed as a randomized, double-blind clinical trial. Participants in this study were collected from the outpatient clinic of the Department of Conservative Dentistry of Medical University of Warsaw (Warsaw, Poland). This study was carried out at the Department of Conservative Dentistry of the Medical University of Warsaw. The study protocol was approved by the institutional bioethics committee (KB/58/2011). All clinical procedures were carried out in accordance with the Helsinki Declaration of 1975, as revised in Tokyo in 2013. Written informed consent was obtained from all participants at the time of being enrolled in the study. This study was registered on ClinicalTrials.gov (NCT06908265, 19 March 2025) “https://clinicaltrials.gov/study/NCT06908265?tab=history (accessed on 19 March 2025)” and was reported in accordance with CONSORT guidelines [23].

### 2.2. Eligibility Criteria

The inclusion and exclusion criteria were selected to keep the study population homogenous and minimize confounding factors that could influence the clinical effects of toothpaste. Participants were eligible for inclusion in the study if they met the following criteria: age between 18 and 65 years, presence of at least 20 teeth excluding third molars, a diagnosis of gingivitis according to the 2017 World Workshop [24], and non-smokers. All participants should have had a motivation to take part in the study and maintain proper oral home hygiene. Written informed consent was obtained from all subjects. The exclusion criteria were as follows: (1) patients younger than 18 years; (2) presence of less than 20 teeth; (3) severe systemic diseases and diseases that require regular systemic drugs (diabetes mellitus); (4) use of systemic antibiotics during the last 3 months or local antiseptics that might affect biofilm formation (antibacterial mouth rinses containing chlorhexidine 4 weeks or less prior recruitment); (5) allergy to mustard or any other compound of experimental toothpaste; (6) ongoing orthodontic treatment; and (7) pregnancy or breastfeeding.

### 2.3. Sample Size Calculation

Assuming a significance level (alpha) of 0.05 and a power of 0.80, the effective size was estimated based on a previous study evaluating a similar intervention [15]. The expected reduction in microbial counts for the experimental group compared to the control group was 30%, and the expected standard deviation was 0.3. Consequently, a sample size of 50 individuals in each of the compared groups was calculated to detect a statistically significant difference between the two groups. In order to account for potential dropouts, 113 subjects were enrolled in this study.

### 2.4. Experimental Toothpaste Preparation

The experimental toothpaste was based on a standard non-fluoride toothpaste formulation with an addition of an extract from white mustard *Sinapis alba*. Briefly, high-quality “Bamberka” type of white mustard seeds were selected and finely ground. The ground seeds were extracted using a Soxhlet extractor made from BORO 3.3 glass according to the DIM 12602 standard (PHU CHEMO-LAB, Ruda Śląska, Poland). This process involved soaking the ground seeds in ethanol, followed by filtration and evaporation of the solvent to yield a concentrated extract [18,19,25]. The high-performance liquid chromatography (HPLC) (Shimadzu, Kyoto, Japan) with UV diode array detector (DAD) (Knauer, Berlin, Germany) methods were used. The extract was then incorporated into the toothpaste base at a concentration of 0.5% by weight. The base formula, a fluoride-free toothpaste without herbal additives, was manufactured by Ziaja LTD (Ziaja ltd Zakład Produkcji Leków sp.z o.o., Gdańsk, Poland). The product was available on the market. The base formula of the toothpaste consisted of common ingredients, such as dicalcium phosphate dihydrate 38%, distilled water 32.4%, glycerol 25%, mustard seeds 5%, hydrated silica 2.4%, carboxymethyl cellulose 1.2%, sodium lauryl sulfate 0.6%, cellulose gum (thickener), essential sodium benzoate 0.2%, and sodium methyl hydroxybenzoate 0.2% [26,27,28]. This product was also considered as a control toothpaste without mustard additives [29].

The stability and quantity of thioglycosides in both toothpaste samples were evaluated with high-performance liquid chromatography (HPLC). After 4 weeks, both the experimental and the control products were dissolved in ethanol at a proportion of 1:20 and placed in 20 mL tubes [30]. A previously calibrated HPLC column with a detector was used to take measurements of retention.

### 2.5. Randomization and Blinding

The participants were randomly assigned to one of the two groups (test or control) using the online computer-generated randomization tool (https://www.randomizer.org accessed on 10 May 2023). The toothpastes were identically packaged and labeled with a code to maintain blinding. The researcher gave the corresponding toothpaste to each patient, with both being blinded to the type of toothpaste.

### 2.6. Study Protocol

**Figure 1:**
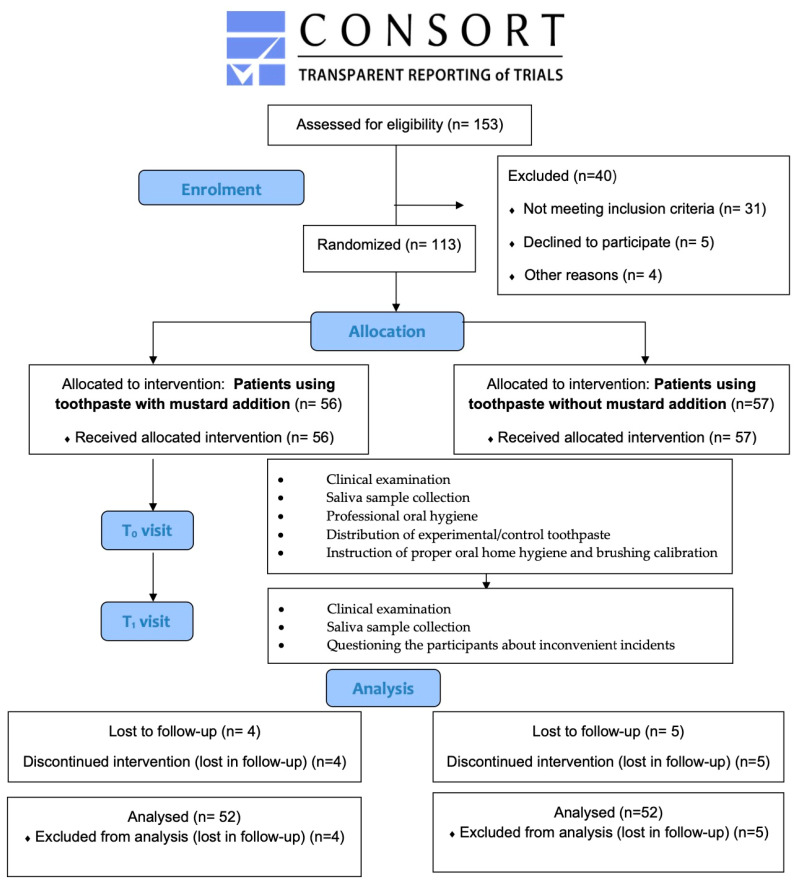
CONSORT diagram showing the study planning.

The subjects for this study were recruited from among patients of the outpatient clinic by a single investigator (A.B.). Patients who met the inclusion criteria were enrolled in this study (Figure 1). At the first visit (T_0_), the clinical evaluation was carried out, and salivary samples for microbial analysis were collected. Subsequently, experimental and control toothpastes were distributed among the patients according to the allocation scheme. The participants were instructed to use a manual toothbrush and brush their teeth using the modified Bass technique. The patients were instructed and trained in this technique at the baseline appointment. All participants were asked to brush their teeth with the assigned toothpaste for 2 min twice a day for 4 weeks.

After 4 weeks (T_1_), the patients were recalled and questioned for any inconvenient incidents during the study period. A clinical examination and the collection of saliva samples were conducted.

### 2.7. Clinical Evaluation

The clinical evaluations were performed by a single masked calibrated examiner (K.M.) at baseline (T_0_) and after 4 weeks (T_1_). A periodontal probe (PCP UNC 15; Hu-Friedy, Chicago, IL, USA) was used to record the following indices:

1. Plaque index (PI) by Silness and Loe [31] was measured on distal-facial, mesial-facial, facial, and lingual sites of all teeth. Presence of plaque at gingival margin was evaluated and the following scores were given: 0—no plaque; 1—a thin layer of plaque only detected by scraping with a probe; 2—moderate accumulation of plaque within gingival pocket, plaque is visible to the naked eye; 3—plaque presence around the gingival margin with vast majority of interdental spaces filled with plaque;
2. Approximal plaque index (API) by Lange [32] evaluated presence or absence of dental plaque in the approximal sides and was calculated as a percentage of the approximal areas that exhibited plaque.
3. Gingival index (GI) by Loe and Silness [33] was measured on six selected teeth (16, 12, 24, 36, 32, 44) on facial, lingual, mesial, and distal sites after gentle probing. The scores were given as follows: 0—normal gingiva, no inflammation, no erythema, no bleeding; 1—mild inflammation, slight erythema, no bleeding; 2—moderate inflammation, erythema, bleeding on probing; 3—severe inflammation, severe erythema and swelling, tendency to spontaneous bleeding.
4. Bleeding on probing (BOP) by Ainamo and Bay [34] was evaluated on facial, lingual, mesial, and distal sites after gentle probing. BoP was calculated by dividing the sum of bleeding sockets by the sum of all evaluated sockets.

### 2.8. Microbiological Analysis

Saliva samples were collected twice from all participants at the beginning of the study (T_0_) and after 4 weeks (T_1_) of using the assigned toothpaste. The samples were collected at least 2 h after the last meal and tooth brushing. A Caries Risk Test (CRT, Ivoclar Vivadent, Schaan, Lichtenstein) was used to evaluate the loads of *S. mutans* and *Lactobacillus* spp. The procedure involved the patient chewing a paraffin pellet to transfer bacteria from tooth surfaces to the saliva, which was then collected in a suitable container. An NaHCO_3_ tablet was placed in the test vial, and upon contact with moisture, it released CO_2_, creating favorable conditions for bacterial growth. After removing the protective foil, the agars were processed quickly to prevent them from standing unprotected for extended periods. Each sample was inoculated using the Ivoclar CRT bacteria test kit, which provides results as either above or below 10^5^ CFU/mL (colony-forming unit/milliliter) for *S. mutans* and *Lactobacillus* spp. The plates were incubated anaerobically at 37 °C for 48 h. After incubation, the plates were evaluated for the growth of bacterial colonies characteristic of the targeted species.

### 2.9. Statistical Analysis

Statistical analysis was carried out using Statistica software, version 13.1 (Statsoft, Kraków, Poland). Descriptive statistics were presented as mean and standard deviation. Any *p*-values of less than 0.05 (*p* < 0.05) were considered statistically significant. A t-test for two independent samples, correlation analysis, and linear regression were employed to thoroughly investigate the relationship between toothpaste use and changes in bacterial loads. The Chi-Square test for independence was conducted to determine if there was a significant association between the type of toothpaste (control vs. experimental) and the loads of *S. mutans* and *Lactobacillus* spp. in saliva.

## 3. Results

One hundred and thirteen patients (55 women and 58 men, aged 28–62; mean age 44.3 ± 16.9 years) were enrolled in this study. In total, 56 subjects were randomly assigned to the experimental group, and 57 subjects were randomized to the control group. Four participants in the experimental group and five participants in the control group were lost in follow-up, and for these reasons, they were excluded from the final evaluation. No adverse or side effects were noted during the study or later.

Between T_0_ and T_1_ in the experimental group, a significant reduction was observed in all four compared parameters: PI (3.68 ± 1.13 to 1.83 ± 0.42, *p* < 0.001), API (41.6 ± 20.5 to 18.5 ± 17.3 *p* < 0.001), GI (1.89 ± 0.76 to 1.45 ± 0.81, *p* = 0.037), and BoP (49.3 ± 15.2 to 18.7 ± 13.6, *p* = 0.017). Similarly, significant results were observed in the control group: PI (3.78 ± 0.72 to 1.25 ± 0.52, *p* ≤ 0.001), API (39.2 ± 19.8 to 22.2 ± 18.6, *p* < 0.001), GI (1.96 ± 0.83 to 1.55 ± 0.92, *p* = 0.043), and BoP (52.9 ± 13.5 to 26.1 ± 16.3 *p* = 0.048).

A statistical analysis containing a t-test for two independent samples showed statistically significant differences between the experimental and control groups and T_o_ vs. T_1_ in the improvement of the PI, API, GI, and BoP. The *p*-values for both tests were well below the significance level of 0.05. The categorized data related to the periodontal parameters and bacteria were presented in Table 1.

**Figure 2:**
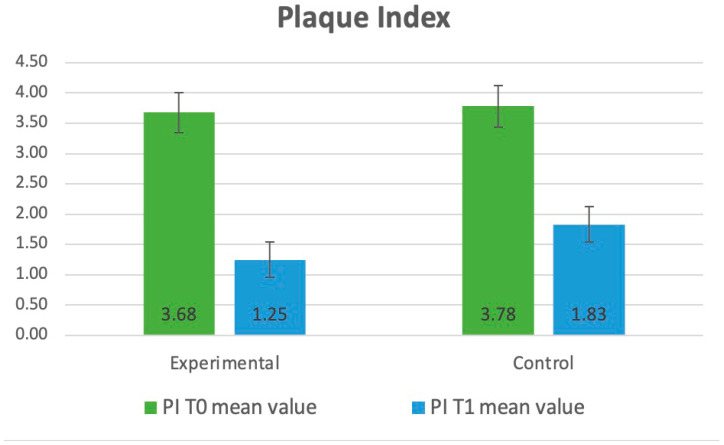
Distribution of the clinical parameters in the groups. Mean values of PI at T_0_ and T_1_.

**Figure 3:**
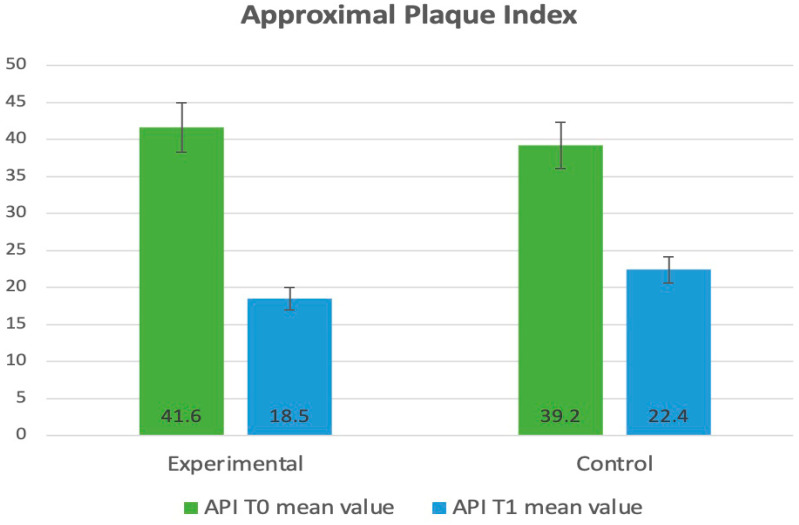
Distribution of the clinical parameters in the groups. Mean values of API at T_0_ and T_1_.

**Figure 4:**
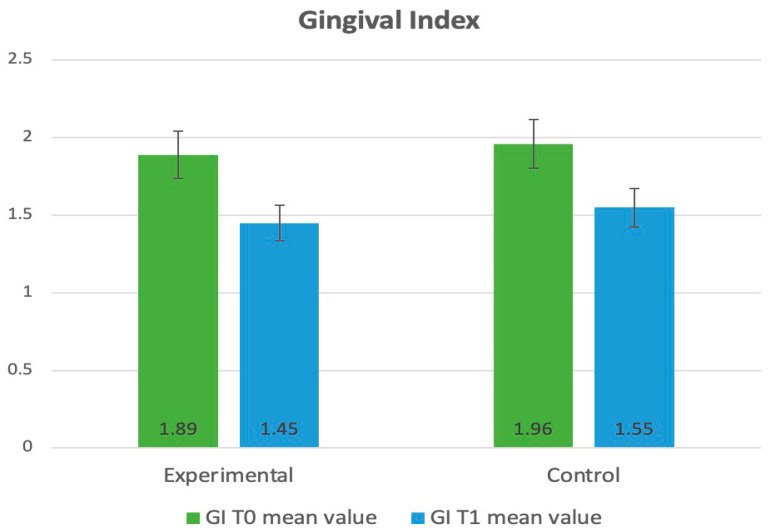
Distribution of the clinical parameters in the groups. Mean values of GI at T_0_ and T_1_.

**Figure 5:**
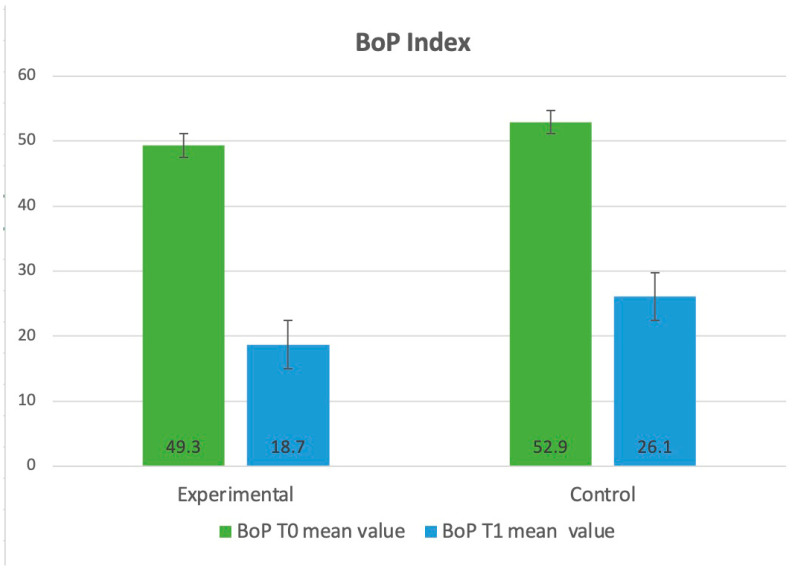
Distribution of the clinical parameters in the groups. Mean values of BoP at T_0_ and T_1_.

The evaluation of clinical parameters (PI, API, GI, BoP) is illustrated and presented in diagrams, as shown in Figure 2, Figure 3, Figure 4 and Figure 5.

At the beginning of the study, 55.8% of the control group samples showed *Lactobacillus* count above 10^5^ CFU/mL. For *Streptococcus mutans*, the control group had an equal distribution, with 50% of samples below 10^5^ CFU/mL and 50% above 10^5^ CFU/mL. In the experimental group, 55.8% of samples showed *Lactobacillus* count above 10^5^ CFU/mL, while for *Streptococcus mutans*, 51.9% were above the 10^5^ CFU/mL level.

After four weeks of using the assigned toothpastes, significant (*p* < 0.001) differences were observed. In the control group, 40.4% of samples showed *Lactobacillus* count above 10^5^ CFU/mL. At the same time, 44.2% of *Streptococcus mutans* samples were above 10^5^ CFU/mL.

In contrast, the experimental group showed a more pronounced reduction in bacterial load. For *Lactobacillus*, only 9.6% was above 10^5^ CFU/mL. For *Streptococcus mutans*, 19.2% samples were above 10^5^ CFU/mL.

**Figure 6:**
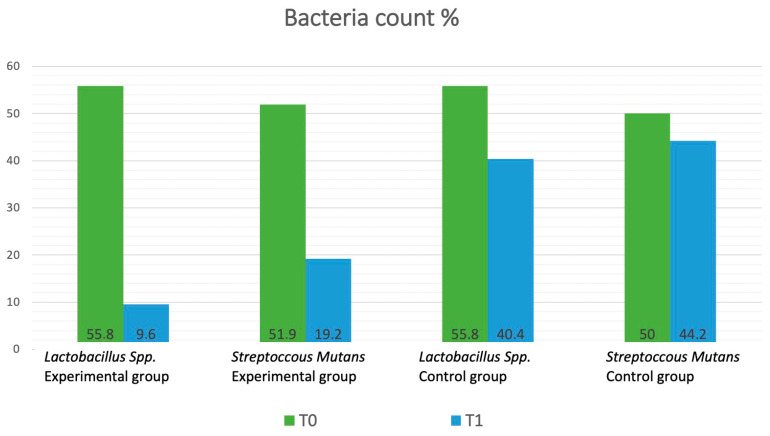
Distribution of the bacteria count in the groups. Percent values of positive (n > 10^5^ CFU/mL) test at T_0_ and T_1_.

Bacteria counts are graphically visualized in Figure 6.

The Chi-Square test for independence was conducted to determine if there was a significant association between the type of toothpaste (control vs. experimental) and the load of cariogenic bacteria *Streptococcus mutans* and *Lactobacillus* spp. The results for *Streptococcus mutans* showed a Chi-Square statistic T_0_—T_1_ (χ^2^) of 10.74 (*p* = 0.000615) and 1 degree of freedom, and for *Lactobacillus* spp., the results showed (χ^2^) 23.12 *p* = 0.000015. These *p*-values are significantly lower than *p* < 0.05, so we assume that the reduction in bacteria count is statistically significant.

## 4. Discussion

The presented study aimed to evaluate the effectiveness of an experimental toothpaste containing white mustard (*Sinapsis alba*) extract in reducing cariogenic bacteria in the oral cavity compared to a standard fluoride-free toothpaste. White mustard was selected for this study due to its well-documented antimicrobial properties, primally attributed to glucosinolates and their hydrolysis products, such as isothiocyanates. These compounds exhibit strong antibacterial effects by disrupting bacterial cell membranes, inhibiting biofilm formation. The findings presented in this study indicate a significant association between the type of toothpaste used and the reduction in bacterial levels, as evidenced by the Chi-Square test results. The experimental toothpaste, enhanced with white mustard extract, showed a marked improvement in reducing both *Streptococcus mutans* and *Lactobacillus* spp. compared to the control toothpaste. This reduction was significant for two key bacterial species responsible for the development of dental caries. The results suggest that the antimicrobial properties of white mustard extract are effective in significantly lowering the levels of cariogenic bacteria. This leads to the conclusion that herbal additives like white mustard extract can be potent agents in oral healthcare. The ability of the experimental toothpaste to eliminate bacteria in a considerable proportion of participants highlights its potential as a preventive measure against dental caries. In addition, this study also revealed significant improvements in clinical parameters related to oral hygiene and periodontal health. These improvements in the PI, API, GI, and BoP suggest that the toothpaste has a positive impact on oral hygiene and decreases the risk of periodontal disease.

The effectiveness of various herbal additives in oral care has been explored, but there is still limited research on the use of mustard-based compounds in toothpaste. However, several studies have investigated the benefits of other plant-based additives, such as tea tree oil, clove oil, and aloe vera, which similarly exhibit antibacterial, anti-inflammatory, and anti-caries properties [28,35]. For example, a study by Arweiler et al. [15] found that toothpaste containing *Scutellaria baicalensis* extract significantly reduced plaque and gingivitis, supporting the potential of herbal additives in promoting oral health. Mustard seed oil properties were also examined by Eichel et al. [19]. This study showed antibacterial effectiveness in an in vitro examination. A recently published study by Michałowski et al. [36] showed an association between using mustard oil extract-based toothpaste and a reduction in periodontal parameters. Literature searches in databases like PubMed and EMBASE did not show any studies linking cariogenic bacteria and mustard oil extract. The concept of incorporating mustard-based compounds in oral hygiene makes our study novel. This is why we cannot compare it to any other clinical study performed previously. This randomized, single-blinded clinical trial provides an adequate evidence level to reduce the knowledge gap in this field.

Fluoride toothpastes are widely recognized as the gold standard in caries prevention due to their ability to enhance enamel remineralization and inhibit demineralization. Numerous studies have demonstrated the efficacy of fluoride in significantly reducing dental caries [37,38]. However, some individuals seek fluoride-free alternatives due to concerns about overexposure or personal health preferences [39]. The current study did not include a direct comparison with fluoride-containing toothpastes, but the significant reduction in bacterial levels observed with the experimental toothpaste suggests that white mustard extract could serve as a potent natural antimicrobial agent. It is worth mentioning that the addition of mustard did not have a significant impact on the taste and odor. None of the participants complained about it.

The literature has not disclosed a clear relation between oral bacteria and mustard oil products. The use of mustard as an additive to oral health products may lead to beneficial results. We would like to address this question in this study. The seed extract preparation process is also typical for the food industry. It proves that it does not lead to a chemical modification of natural contents. The concentration of active ingredients in the prepared formulations was lower than in food mustard [40].

This study had several limitations. The short duration of the study—four weeks—does not allow for a comprehensive evaluation of the long-term effects on oral health. However, the four-week study duration was chosen because bacterial levels in saliva change rapidly, with measurable reduction occurring within days of antimicrobial intervention. This period allows for assessing both short-term antibacterial effects and clinical improvements while ensuring participant compliance. A longer duration increases the number of dropouts and additional variables. This period provides a practical and controlled evaluation. Previously conducted studies on herbal additives in toothpaste like chitosan lasted four weeks [41].

The sample size, while sufficient for initial findings, could be expanded in future studies to increase statistical power and generalizability. The lack of comparison with fluoride-containing toothpastes is another limitation, as fluoride remains the most well-researched and effective agent in caries prevention. A direct comparison of mustard extract with fluoride would provide valuable insights into the relative efficacy of natural alternatives. Another potential limitation is the allergenic potential of mustard seeds. Although no allergic reactions were reported during this study, mustard seed is known for its high allergic potential [42,43]. Only external use was applied in this study. For these reasons, no preliminary toxicological studies were performed.

Future studies should explore the mechanisms by which white mustard extract demonstrates its antibacterial effects. Further research should investigate the optimal concentration of white mustard extract and its combination with other herbal additives to maximize its antimicrobial properties. Researchers should focus on long-term studies that assess the sustained effectiveness of white mustard toothpaste in reducing cariogenic bacteria and preventing caries. Additionally, studies that directly compare mustard-based toothpaste with fluoride-containing formulations would provide a more comprehensive understanding of its efficacy. A comparison of mustard-based toothpaste to other plant-based would be beneficial. Investigating the optimal concentration of mustard extract and its potential synergistic effects with other natural compounds, such as xylitol or tea tree oil, could further enhance its antibacterial properties. Moreover, exploring the broader impact of mustard extract on the oral microbiome, including its effects on non-cariogenic species and overall microbial diversity, would provide valuable insights into its role in promoting oral health.

The results suggest that regular use of herbal toothpaste with mustard extract could contribute to caries prevention, offering a natural alternative for individuals seeking non-fluoride solutions.

## 5. Conclusions

This study demonstrates that toothpaste enhanced with white mustard extract is significantly more effective in reducing cariogenic bacteria, such as *Streptococcus mutans* and *Lactobacillus* spp., compared to a fluoride-free control toothpaste. Continued research in this area could lead to the development of more effective and natural oral hygiene products, ultimately expanding the options available for maintaining oral health in diverse populations. Further research is recommended to confirm long-term benefits, assess additional health effects, and analyze the potential of these toothpastes to prevent dental caries.

## Figures and Tables

**Table 1:** Distribution of the results in the groups. Mean values of PI, API, GI, BoP, and bacteria load for number of samples and a paired t-test in the experimental and control groups at T_0_ and T_1_.

	Time	Experimental	Control	*p*-Value Exp. vs. Contr.
**Anamnestic Data** Age (mean) Gender		43.2 ± 12.2 years 25 F/31 M	45.9 ± 16.2 years 30 F/27 M	
**PI Mean (SD)** T_o_ vs. T_1_	T_0_ T_1_	3.68 (1.13) 1.25 (0.42) *p <* 0.001	3.78 (0.72) 1.83 (0.52) *p <* 0.001	*p* = 0.599 *p =* 0.041
**API Mean (SD)** T_0_ vs. T_1_	T_0_ T_1_	41.6 (20.5) 18.5 (17.3) *p <* 0.001	39.2 (19.8) 22.4 (18.6) *p <* 0.001	*p =* 0.692 *p* = 0.846
**GI Mean (SD)** T_0_ vs. T_1_	T_0_ T_1_	1.89 (0.76) 1.45 (0.81) *p =* 0.037	1.96 (0.83) 1.55 (0.92) *p =* 0.043	*p =* 0.661 *p =* 0.565
**BoP Mean (SD)** T_0_ vs. T_1_	T_0_ T_1_	49.3 (15.2) 18.7 (13.6) *p =* 0.017	52.9 (13.5) 26.1(16.3) *p* = 0.048	*p* = 0.675 *p =* 0.037
***Streptococcus mutans* Bacteria Load** T_0_ vs. T_1_	T_0_ T_1_	27/52 (n > 10^5^ CFU/mL) (51,9%) 10/52 (n > 10^5^ CFU/mL) (19,2%) *p =* 0.012	26/52 (n > 10^5^ CFU/mL) (50%) 23/52 (n > 10^5^ CFU/mL) (44.2%) *p* = 0.694	*p* = 0.9876 *p* = 0.011
***Lactobacillus*** spp. **Bacteria Load** T_0_ vs. T_1_	T_0_ T_1_	29/52 (n > 10^5^ CFU/mL) (55.8%) 5/52 (n > 10^5^ CFU/mL) (9.6%) *p* < 0.001	29/52 (n > 10^5^ CFU/mL) (55.8%) 21/52 (n > 10^5^ CFU/mL) (40.4%) *p* = 0.169	*p =* 1 *p* < 0.001

T_0_,T_1_—time frames; M—male; F—female; BoP—bleeding on probing; PI—plaque index; GI—gingival index; API—approximal plaque index; SD—standard deviation N—number of samples; n—number of bacteria colony; CFU—colony-forming unit.

## Data Availability

The data presented in this study are available on request from the corresponding author.

## Abbreviations

The following abbreviations are used in this manuscript:

PI	Plaque Index
GI	Gingival Index
API	Approximal Plaque Index
BoP	Bleeding on probing
